# Determination of the Volatile Composition of *Rhodobryum giganteum* (Schwaegr.) Par. (Bryaceae) Using Solid-phase Microextraction and Gas Chromatography/Mass Spectrometry (GC/MS) 

**DOI:** 10.3390/molecules14062195

**Published:** 2009-06-17

**Authors:** Lin Li, Jiancheng Zhao

**Affiliations:** College of Life Science, Hebei Normal University, Shijiazhuang, 050016, China; E-mail: violi2005@126.com

**Keywords:** *Rhodobryum giganteum* (Schwaegr.) Par., HS-SPME, GC/MS, volatile

## Abstract

A total of 38 volatile components were identified in *Rhodobryum giganteum* (Schwaegr.) Par. collected from two different geographic regions by headspace solid-phase microextraction (HS-SPME), combined with gas chromatography/mass spectrometry (GC/MS). The volatile components included some aliphatic and aromatic aldehydes, monoterpene hydrocarbons and a sesquiterpene (α-farnesene), with 1-methoxy-2-propyl acetate and *n*-hexanal being found to be the most abundant volatile components. Analysis of the chemical constituents in the volatile oil of the two samples showed that ten compounds were shared.

## 1. Introduction

Bryophytes constitute a special group of higher plants which are known to possess various and novel natural products, many of which exhibit interesting biological activities such as antibacterial, antitumor, antiseptic, anticoagulant, insect antifeedant, nerve protection and cytotoxic properties [1,2,3,4]. Bryophytes include the Hepaticae (liverworts), Anthocerotae (hornworts) and Musci (mosses). The chemistry of liverwort, which is a coordinate phyla paralleled with moss, has been the subject of intensive investigations, mainly because liverworts contain cellular oil bodies and produce a number of terpenoids, aromatic compounds and acetogenins, several of which show interesting biological activities such as allergenic contact dermatitis, insecticidal, insect antifeedant, cytotoxic, piscicidal, muscle relaxing, plant growth regulatory, anti-HIV, DNA polymerase beta inhibitory, anti-obesity, neurotrophic, NO production inhibitory, antimicrobial and antifungal activities [5,6,7,8].

Interestingly, much less interest has been devoted to the mosses, despite the fact they are more widely distributed than that of liverworts, although since ancient times, several species of mosses have been used as herb medicine to treat trauma, empyrosis, infestation, pulmonary phthisis, eclampsia, pneumonia, and so on. *Rhodobryum giganteum* (Hook.) Par. and *Rh. roseum* (Hedw.) Limpr. from the Bryaceae moss family, are known as “Huixincao herb” in folk medicine in different areas of Yunnan province (P.R. China), especially by many of the ethnic minority groups, such as the Yi, Dai and Hani nationalities [9,10]. The herb is used for the treatment of heart problems, nervous prostration and cardio-vascular diseases [11,12,13,14,15].

*Rh. giganteum* is mainly distributed in China (the south regions of the Yangtze River), Korea, Japan, south India and other Southeast Asia regions. The species is about 3-6 cm tall and grows on humus soil and logs in forest shade and has a distinctive characteristic of biseriate teeth in middle-upper leaf margin. *Rh. roseum*, with a smaller size about 1-3 cm tall and uniseriate teeth in middle-upper leaf margin, is found widely distributed in China, Europe, central and south Africa, North America and Mexico [16]. As medicinal materials, these plants are very important for their chemical composition. Phytochemical studies have identified the presence of alkaloid, sterol, triterpenes, ursane terpenoid, diterpene glycoside, phenolic acid, fatty acid, biphenyl and carbohydrate in *Rh. Roseum* [17,18,19]. However, chemical composition of *Rh. giganteum* has not been comprehensively analyzed.

The volatile oil constituents of *Rh. giganteum* from different geographical localities have not been documented in the literature, although a previous analysis using hydrodistillation to extract volatiles from the species has been reported, enabling the identification of several types of compounds, mainly organic acids, esters, alkenes, aldehydes and alcohols [20]. Hydrodistillation, which is a traditional extraction methodology, has been applied to essential oil extraction from plant materials, although it presents some shortcomings, such as losses of volatile compounds, low extraction efficiency and long extraction times. Also, the high temperatures and water can cause degradation or chemical modifications of some volatile constituents [21].

In recent decades, the most frequent analytical techniques applied in the extraction and concentration of volatile compounds from aromatic and medicinal plants are those based on headspace analysis (HS). Among the headspace methods, the solid-phase microextraction (SPME) constitutes a reliable tool for the analysis of organic volatile and also semi-volatile compounds. The aim of this work was to extend the knowledge of volatile compounds of *Rh. giganteum* collected from different geographic regions by using the HS-SPME technique.

## 2. Results and Discussion

The results of the SPME-GC/MS analysis of the composition of the two samples of *Rh. giganteum* are presented in Table 1.

**Table 1:** Chemical composition of two samples of *Rh. giganteum* (Bryaceae) and their corresponding retention indices (RI).

Compounds	RI	Area ( %)^a^	
		S1	S2
Pyridine	740	7.43	4.15
2H-Pyran,3,4-dihydro-	748	-^b^	4.08
Dimethylformamide	783	7.52	1.95
*n*-Hexanal	804	8.55	19.67
2-Hexenal	861	-	1.33
1-Methoxy-2-propyl acetate	870	8.44	7.89
Isohexanol	873	-	3.91
1-Hexanol	875	3.45	-
Diethylcyanamide	903	-	0.99
*n*-Heptanal	906	-	3.10
α-Pinene	943	-	1.04
Benzaldehyde	974	-	0.84
Amyl vinyl carbinol	984	-	1.20
6-Methyl-5-heptene-2-one	990	3.14	2.51
2-Pentylfuran	994	-	0.90
Caprylic aldehyde	1008	1.64	1.55
2-Ethylhexanol	1035	1.66	-
Limonene	1039	1.18	6.69
*n*-Undecane	1100	-	1.11
*n*-Nonanal	1109	4.61	4.04
Camphor	1161	4.14	0.98
Tetralin	1174	-	1.36
*n*-Decanal	1210	3.18	1.19
Hexyl isovalerate	1238	-	0.76
Butyric acid, 2-methyl-, hexyl ester	1240	1.80	-
2-Ethyl-5-methyl-furan,	1258	0.71	-
Estragole	1295	-	7.37
Lauraldehyde	1414	1.47	-
1,4-Methanoazulene, decahydro-4,8,8-trimethyl-9-methylene-,[1s-(1α,3αβ,4α,8αβ)]-	1430	1.73	-
Geranylacetone	1453	2.76	-
Pentadecane	1501	2.29	-
Farnesene	1508	2.13	-
Butylated hydroxytoluene	1510	-	1.29
*n*-Tridecylaldehyde	1516	3.33	0.75
*n*-Hexadecane	1600	5.45	1.23
Crocetane	1648	2.78	-
*n*-Heptadecane	1700	-	1.53
2,6,10,14-Tetramethylpentadecane	1701	8.02	-
Total identified	-	87.41	83.41

^a^ percentages of the peak area relative to the total peak area of the identified compounds; ^b^ not detected.

A total of 38 volatile components were identified in the two samples. It is apparent from the data shown that the components of the two samples of *Rh. giganteum* were abundant in aliphatic and aromatic aldehydes (*n*-heptanal, *n*-nonanal, *n*-decanal, benzaldehyde, 3,4-dimethylbenzaldehyde, lauraldehyde) and alkanes (*n*-undecane, *n*-pentadecane, *n*-hexadecane, *n*-heptadecane). In addition, a few terpenoid compounds were detected. The monoterpene hydrocarbons α-pinene and limonene, as well as camphor, which are very common volatile compounds of mosses were present. A sesquiterpene hydrocarbon, namely α-farnesene, was only detected in sample 1 (S1).

Thirty-one components were detected in S1, of which twenty-three components representing 87.41% of the total volatile, were identified. According to our results, the major constituents were *n*-hexanal (8.55%), 1-methoxy-2-propyl acetate (8.44%), 2,6,10,14-tetramethylpentadecane (8.02%), *n*-hexadecane (5.45%), nonanal (4.61%) and camphor (4.14%). These components account for 39.21 percent of the total volatile composition while the other minor components make up the balance.

Out of forty-one peaks detected in Sample 2 (S2), twenty-seven components, which accounted for 83.41%, were identified in the volatile oil. The oil derived from S2 is dominated by *n*-hexanal (19.67%), 1-methoxy-2-propyl acetate (7.89%), estragole (7.37%), limonene (6.69%), 3,4-dihydro-2*H*-pyran (4.08%) and nonanal (4.04%).

Our analysis of the chemical constituents in the volatile oil in the two samples of *Rh. giganteum* showed similarity in their chemical composition. Ten compounds, which accounted for 43.66% and 46.50% of the total volatiles, respectively, were common to the two samples, and *n*-hexanal and 1-methoxy-2-propyl acetate have been identified as main constituents in both of them. Aromatic aldehydes dominated in two samples of the species, separately accounting for 22.78% and 32.47% of the total. The percentages of the peak area relative to the total peak area of the identified compounds were also given in Table 1, and these data demonstrate the composition was different not only quantitatively but also qualitatively. For instance, estragole, which accounted for 7.37% of the volatile oil of S2 as main constituent, was not present in S1. The chemical composition differences of the two samples might be caused by the ecological niches, climatic conditions and other biotic factors. The presence of pyridine and dimethylformamide in the samples does not seem appropriate. These two compounds could be solvents used in the laboratory and they were absorbed by the fiber of HS-SPME. They were ignored as dominant and shared compounds in the volatiles of the two samples.

## 3. Experimental Section

### 3.1. Plant Materials

Samples of *Rh. giganteum* were separately collected from the Gaoligongshan National Natural Reserve in Lushui county, Yunnan Province, China, with the geographical coordinate of 26°00′N and 98°39′E on humus soil in October 2008 (S1) and Liufengshan Mt. in Kaiyuan city, Yunnan Province, China, with the geographical coordinate of 23°02′N and 103°11′E in forest shade on humus soil in February 2009 (S2) and identified by Prof. J.C. Zhao. Voucher specimens were deposited in the Herbarium of College of Life Science, Hebei Normal University (HBNU), Shijiazhuang, China.

### 3.2. Sample preparation and analysis by HS-SPME

The pulverized, silica gel dried, 1.0 g S1/S2 powder was weighed and immediately introduced into a 25 mL headspace vial. A fused-silica fiber coated with a 75 μm layer of carboxen/polydimethylsiloxane (CAR/PDMS) was used for absorption of the volatile compounds. The fiber was then exposed to the headspace in the vial at 25 °C for 30 min and then the SPME device was removed from the vial and inserted into the injector port of the GC system for thermal desorption which was performed 3 min at 250 °C.

### 3.3. Gas chromatography-mass spectrometry

A Thermo Focus DSQ gas chromatography-mass spectrometry was used in the study. Chromatography was performed on a VF-5 ms capillary column (30 m ×0.25 mm; 0.25 μm film thickness). Helium was used as the carrier gas with a flow rate of 1.0 mL/min. The injection and ion source temperatures were both 250 °C. The GC oven temperature was programmed from 60 °C, held 2 min, raised to 300 °C at 10°C/min, and held for 10 min. The electron impact technique of 70 eV was used and the mass range scanned was 41-450 amu in full-scan acquisition mode. The identification of the compounds was based on comparison of their retention indexes (RI), obtained using n-alkanes (C7-C21), and retention times. Compounds were also confirmed by comparison of their mass spectra with the NIST/NBS-Wiley library spectra. The relative amounts of individual components of the volatiles of the two samples were expressed as percentages of the peak area relative to the total peak area. Relative percentage amounts were calculated from the TIC by the computer. 

## 4. Conclusions

Analyses of the volatile constituents from the plants of *Rh. giganteum* collected from different locations indicate *n*-hexanal and 1-methoxy-2-propyl acetate to be the predominant components of *Rh. giganteum*. This work provides the first report of the analysis of *Rh. giganteum* from different habitats in southwest China.
